# Risk of Upper Extremity Injury in Recreational Pickleball Players

**DOI:** 10.3390/jfmk10030247

**Published:** 2025-06-28

**Authors:** June Hanks, Betsy Myers

**Affiliations:** Department of Physical Therapy, College of Health, Education and Professional Studies, University of Tennessee at Chattanooga, Chattanooga, TN 37403, USA; betsy-myers@utc.edu

**Keywords:** pickleball, injury, upper extremity, risk, play volume, grip, skill level

## Abstract

**Background:** With the increasing popularity of pickleball (PB) has come an increase in upper extremity (UE) injury. This study examines the relationship between PB-related UE injury and player characteristics, typical weekly playing behavior, grip tightness, and stretching or strengthening exercise among recreational PB players. **Methods:** Players at least 18 years of age who played PB for at least six months were recruited to complete an anonymous online survey. **Results:** Among the 253 participants, 41% reported at least one UE injury: 10% acute and 37.5% chronic. Chi-square analysis (α = 0.05) was used to determine between group differences. The risk of UE injury was 1.51 to 1.53 times higher among individuals who played longer or more frequent sessions or played on consecutive days. Acute injury was more likely in those who played >two hours at a time, on consecutive days, or maintained a tight baseline grip—with relative risks of 2.38, 4.97, and 2.67, respectively. Chronic injury was more common in those who played >two years, at higher skill level, or >six hours a week. No difference in UE injury and sex, strengthening, or stretching was found. **Conclusions:** The risk of UE injury was higher for those who play longer, more frequently, or on consecutive days. Additionally, acute injury was more likely when using a tight grip for baseline shots and chronic injury was more common among those who played more years, at a higher skill level, or greater playing volume.

## 1. Introduction

Originating near Seattle, Washington in 1965, pickleball (PB) is now one of the fastest-growing sports in the United States (US) with players spanning all age groups [1]. Though PB shares similarities with tennis, badminton, and paddleball, key differences exist in court dimensions, player positioning, and equipment (racket/paddle and ball/shuttlecock). Additional distinctions include the shorter learning curve to become competitive and the lower physical fitness and agility requirements of players. Initially popular among older adults, the sport’s demographic is shifting. In 2025, adults aged 25–34 constitute the largest segment of PB players, with the average player age being 34.8 years [2]. As PB play has surged, so too has the number of injuries [3]. Alarmingly, the rate of PB-related injuries is increasing more rapidly than the rate of participation [3,4]. Because PB is a young sport, there is less published literature on PB-related injury compared to more established racket sports such as tennis and badminton.

Consistent with epidemiological studies of other racket or paddle sports [5,6], playing PB poses a risk for acute upper extremity (UE) injury such as strain/sprain, or fracture [7,8,9], and chronic UE injury, such as epicondylalgia [10,11]. Analysis of US emergency department (ED) data indicates PB-related injury to the UE in approximately in 25–33% of patients, with an increasing number of injuries across the study periods [7,8]. A high number of UE PB-related injuries treated in locations other than the ED have also been reported [3,12].

Sex differences in injury patterns have also been noted. In badminton, males are more commonly injured than females, however injury rates were the same when accounting for playing time [6]. In PB, women are more likely to experience fractures [13], particularly of the wrist [3,8]. Age is a well-documented risk factor for racket/paddle-sport-related injuries, with several studies reporting higher injury rates among older players [14,15], though not all studies differentiated between UE and LE injury. Data from the National Electronic Injury Surveillance System (NEISS) confirms that racket/paddle sport-related injury is more common among older individuals [4,7,16]. However, injury risk is not exclusive to older populations. With the increasing participation in PB among younger adults, injury rates in these age groups are also rising [4].

Playing behavior, such as high weekly frequency of play, long session lengths, and greater years of play, poses a higher risk of a chronic UE injury in tennis [17,18] and padel players [19]. In tennis, grip tightness during and following ball contact has been related to higher forces to UE, particularly during off-center impacts [20,21] and during backhand strokes [22].

Stretching and strengthening to facilitate recovery from UE injury is well supported in the literature [2,3,4,5,6,7,8,9,10,11,12,13,14,15,16,17,18,19,20,21,22,23,24,25]. However, the link between UE injury risk and participation in strength and flexibility exercises is unclear. While some studies find no benefit to these types of training [26], more recent studies indicate that strengthening and stretching can reduce injury rates, especially in overhead sports [14,27].

This study examines the relationship between UE injuries and recreational PB player characteristics, playing behavior, grip tightness, and participation in strengthening and stretching exercises. Findings will help inform both players and healthcare providers regarding evidence-based prevention strategies for upper extremity injuries in PB.

## 2. Materials and Methods

### 2.1. Participants

An invitation to participate in an anonymous online survey using the Qualtrics survey platform was posted at multiple recreational facilities with PB courts (both indoor and outdoor) and on various PB-related social media group sites across the US. Additionally, PB group social media leaders were asked to share the survey with their internal email lists. The survey could be completed on a computer, tablet, or mobile phone. Participation was voluntary.

To enter the survey, participants had to confirm they were at least 18 years old and had played PB for at least six months. The survey included questions related to sex, age, skill level, number of years playing PB, and typical playing behavior. Participants also reported perceived grip tightness during dinks, volleys, and baseline hits, and whether they engaged in an upper extremity (UE) strengthening program—using free weights, body weight exercises, resistance machines, or other methods—or a stretching program, including yoga (instructor-led or self-guided), general stretching (instructor-led or self-guided), or other forms, at least 1–2 times per week. Skill level definitions, as published by the USA Pickleball Organization [28], were provided within the survey. Participants reported whether they had experienced an acute UE injury, a chronic UE injury, and which anatomical region (shoulder, elbow, wrist/hand) had been injured. In the survey, acute injury was defined as an acute/sudden injury, such as fracture, sprain or strain that occurs due to a sudden identifiable traumatic event, such as a wrist fracture from falling or ankle sprain from stepping on a ball. Chronic injury was described as injury that occurs gradually over time due to overuse, with worsening pain or discomfort during or after playing and becoming worse with continued use. The survey remained open for six months. This study was approved by the Institutional Review Board of the University of Tennessee at Chattanooga.

### 2.2. Statistical Analysis

Survey results were imported into SPSS version 29.0.2. Unanswered questions were coded as missing and excluded from the analysis. A descriptive analysis was performed to obtain information regarding the number of times the categories of each study variable occurred (frequency and percentage). The chi-squared test of independence (α = 0.05) was used to determine differences in the report of at least one acute or chronic UE injury and the following variables: sex, age, skill levels of play, and playing behavior (number and length of sessions per week), consecutive days of play, and overall play volume). Additional analyzed variables included perceived grip tightness measured on a 5-point Likert scale during dinks, volleys and baseline play; as well as engagement in UE strengthening or stretching exercises at least once or twice a week. The association between skill level and playing behavior was calculated. Effect sizes were determined for all significant differences using phi for dichotomous variables and Cramér’s V for non-dichotomous variables. The magnitude of effect for both phi and Cramér’s V was classified as small (0.1), moderate (0.3), or large (0.5) [29].

For significant associations involving three categories, post hoc tests were performed using the Bonferroni correction to control for the risk of type I errors. The adjusted *p*-value for post hoc testing was calculated as 0.05/3 = 0.0167. Due to sample size considerations, dichotomous categories were created for all variables except age. Age was grouped into three categories to allow comparisons of younger adults (18–34 years old), middle-aged adults (35–64 years old), and older adults (≥65 years old). Cut points for non-dichotomous variables (years of play, session length, and play volume) were based on mean responses approximating a 50% split. While the average number of times played per week reported in this sample was 3.7, a cutoff of 3 (i.e., ≤3 vs. ≥4) was chosen because those playing 4 days per week must play on consecutive days while those who play 3 times per week may not, thus potentially differentiating between these two groups. Skill level was collapsed into two categories (≤3.5 vs. >3.5) to reflect differences in consistency, strategy, shot selection, and control that mark the transition to the 4.0 skill level. Because a firmer grip may be linked to injury, perceived grip was grouped into two categories: sufficient and tight, with sufficient being defined as “just tight enough to control the paddle at the time of ball contact” and tight defined as “as tight as possible”.

## 3. Results

### 3.1. Survey Participation and Demographics

A total of 269 participants began the survey and 253 participants (94%) completed it. Data from the 253 participants were included in the final analysis. See Table 1 for frequency data of the sample. The average age of participants was 52.8 years. One hundred and four participants reported a total of 152 UE injuries. There were 30 acute injuries: 16 strain/sprains (14 shoulder, two wrist/hand), 10 fractures (four elbow, six wrist/hand), and four unspecified (three shoulder, one elbow). There were 122 chronic injuries. Three participants reported more than one acute injury, while 18 participants report more than one chronic UE injury.

**Figure 1:**
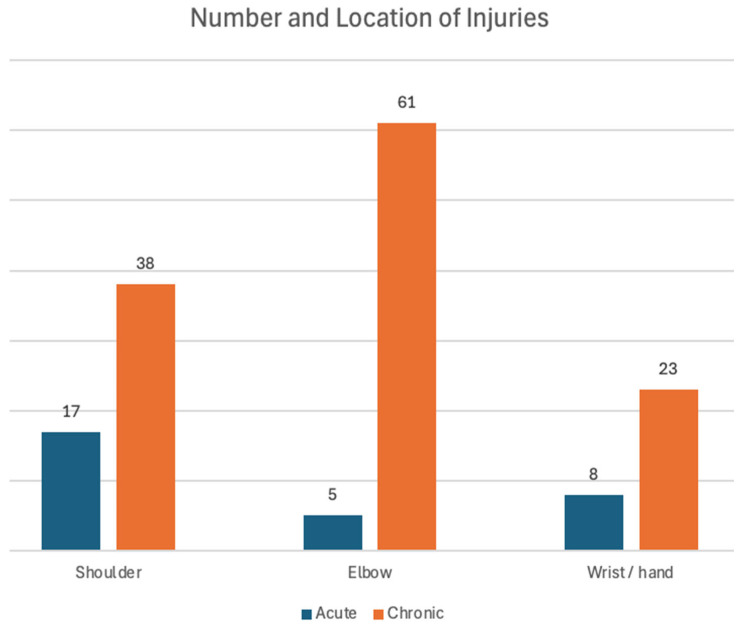
Number and location of injuries by type.

Figure 1 provides details on acute and chronic injury location. Of the 61 reported chronic elbow injuries, 38 were lateral, 13 were medial, and 10 were both lateral and medial. Among participants who reported engaging in strengthening exercises, 97 utilized free weights, 79 performed body weight resistance exercises, and 49 used resistance machines. Of participants reported engaging in stretching activities, 114 performed general stretching and 29 performed yoga.

### 3.2. Overall Upper Extremity Injury

Overall UE injury was not significantly different based on sex. Therefore, data from both sexes were pooled for analyses. The average age of participants was 52.8 years (SD = 16.7; range: 19–83 years). Differences between participants reporting an injury and not reporting an injury are reported in Table 2. There were significant differences with small effect in reported UE injury based on age, session length, play frequency, and playing on consecutive days. A post hoc analysis of age indicated the middle age group is significantly different than the younger or older age groups, with the middle age group reporting more UE injuries. In participants with UE injury there was an association of small to moderate effect between skill level and the play behaviors of sessions played per week [Χ^2^(1) = 11.947, *p* = 0.005, phi = 0.219], play volume per week [Χ^2^(1) = 13.014, *p* = 0.003, phi = 0.229], and playing on consecutive days [Χ^2^(1) = 13.014, *p* = 0.003, phi = 0.229], and no association between skill level and session length [Χ^2^(1) = 3.352, *p* = 0.067]. Higher-skilled players were significantly more likely to play more frequently each week, spend over six hours playing, and participate on consecutive days compared to players with lower skill levels.

### 3.3. Acute Upper Extremity Injury

Acute UE injury was not significantly different based on sex, so data from both sexes were pooled for subsequent analyses. Between groups difference in reported acute injury are in Table 3. There were significant differences with small effect in reported acute UE injury based on session length, playing on consecutive days, and perceived grip tightness during baseline shots. No significant between group differences were found for demographic factors, other playing characteristics, or grip tightness during dinks or volleys.

### 3.4. Chronic Upper Extremity Injury

There was no difference in chronic UE injury and sex, therefore, data from both sexes were pooled in the analysis. Chronic UE between group differences are reported in Table 4. The report of a chronic UE injury was significantly associated with age, with middle-aged individuals more often reporting a chronic UE injury than the younger or older participants, with all differences of small effect. Those who have played for more years had a higher skill level, played longer or more frequent sessions, played a greater volume, or played on consecutive days were significantly more likely to report a chronic UE injury. No significant differences were found between the report of a chronic UE injury and perceived grip tightness, or participation in stretching or strengthening programs.

### 3.5. Relative Risk for Injury

The relative risk for a UE injury (overall, acute, and chronic) is reported for each significant variable in Table 5.

## 4. Discussion

### 4.1. General Overview

Among participants, 51% were in the 35–64-year-old age category, with participants ≥ 65 years of age or older and players 18–34 years of age representing the remainder of participants at 27% and 22%, respectively. The average age of participants in this study is higher than the reported average player age of 35 years [2], which may be explained by the inclusion criteria of PB play for at least six months and a minimum age of 18 years. Overall, the age of participants in this study reflects the recent increasing number of younger individuals playing PB, a sport initially played primarily by older adults. In this study, fewer than 25% of participants reported a skill level of 4.0 or greater.While the skill level of recreational players has not been previously reported, the lack of highly skilled players in this study suggests participants adequately reflect the characteristics of a recreational player.

Playing PB poses a risk of UE injury. In the present study, 41% of participants self-reported an acute or chronic UE injury which is comparable to the 39% found by Myers and Hanks [10], and the 36.5% in recreational padel players (36.5%) found by Thomas et al. [20]. In contrast, data from ED databases show lower rates of UE injury, ranging from 26% [7] to 31% [3]. This discrepancy may be due to an underreporting in ED data, given that individuals with less severe injuries are less likely to seek urgent care.

In this study, an acute UE injury was 2.38 times more likely for those playing 2 h or more hours per session and nearly five times more likely for those playing on consecutive days. The fatigue associated with playing longer sessions and on consecutive days may contribute to an acute injury. Using a tight grip during baseline shots resulted in a nearly three-fold increase in acute injury. In tennis players, differences in muscle activation during the various phases of the two-handed volley and groundstrokes are reported, with more wrist stabilization required during the volley than groundstroke [30]. These findings should not immediately be generalized to PB, due to differences in the tennis racket and PB. Additionally, the present study did not differentiate one versus two handed shot for the forehand or backhand. Also, the reported grip was a perceived, not measured grip. Though a tighter grip than necessary to control the paddle at ball contact may result in muscle activation so great that the risk of an acute injury is elevated, more research is necessary to explain injury risk related to grip tightness.

This study found 37.5% of participants reported a chronic UE injury, a finding consistent with the 39% found in a separate study of recreational PB players [10], but lower than the 63.2% reported among club tennis players [5]. The lighter pickleball, using a shorter paddle and with a shorter shot distance, might explain these differences.

### 4.2. Sex and Age

Sex was not different between those reporting or not reporting a UE injury. This is similar to studies in tennis [31,32,33] and badminton [6]. Middle-aged participants were more likely report a UE injury in general, and a chronic UE injury in particular. Using ED data, Changstrom [16] found a mean age of 37 years for non-PB racket/paddle-related injuries, while Forrester reported a mean age of 63 years for PB-related injuries [7]. However, neither study differentiated UE injuries by age. Among club tennis players, the prevalence of lateral epicondylagia was higher among middle-aged players than younger players and leveled off among older players [17]. It is possible that older PB players are more likely to stop play or reduce play frequency.

### 4.3. Skill Level and Play Behavior

In this study, a chronic UE injury was more common in the more highly skilled PB players. While Jørgensen [6] found no difference in overall injury rates between elite and recreational players, overuse injuries—particularly to the upper extremity—were significantly more common in elite players. There appears to be an interaction between skill level and play behaviors. Overall, higher-skilled players played more frequently, played longer sessions, and played on consecutive days compared to players with lower skill levels. These findings align with injury and play behavior in other racket/paddle sports, with a study of tennis players reporting tennis elbow pain among those playing 3.3 games/week and 8 h of play/week compared to a report of no tennis elbow pain among those playing 2.9 games/week and 5.6 h/week [17]. In padel players, a higher UE injury risk occurred among those who had higher playing volume [19]. Intuitively, one might expect that advanced players use superior techniques, which would reduce their injury risk. Indeed, studies of tennis players have demonstrated that advanced players utilized techniques that reduced joint upper extremity forces [22,33]. While increased playtime may foster skill development, it is also associated with a higher risk of chronic UE injury [17]. Therefore, it is plausible that volume of play, rather than skill level alone, exerts a critical role in the development of chronic UE injury among pickleball players. Further research is warranted to better understand the interplay between skill level, play volume, and injury risk.

### 4.4. Stretching and Strengthening

Over half (52.6%) of participants reported engagement in UE strengthening or stretching exercises at least 1–2 times per week, but no difference was found between participation and the report of UE injury. In this study, the most common UE strengthening and stretching exercises were free weights and general stretching, respectively. Reduced muscle length and joint range of motion (ROM) are associated with a greater injury risk [34]. Strengthening exercise should be tailored to meet sport-specific requirements [35]. While strengthening and stretching exercises are commonly recommended as part of training programs for racket/paddle sports players [36], the specific type and volume of strengthening and stretching exercise required for UE injury reduction has not been specified for PB.

### 4.5. Grip and Injury

The association of a tight baseline grip with acute UE injury was not surprising, but the lack of association with chronic injury was unexpected. Previous work has linked grip tightness with chronic injury in other racquet sports [20]. It is possible that the mechanics of pickleball, with its lighter ball and shorter racket, creates less UE tissue stress than tennis. Perhaps in PB, factors other than grip tightness, such as spin and grip pattern, influence UE injury. Future studies should investigate these variables.

A limitation in this study is self-reporting bias with the potential for participants to under- or over-report injury. Although the definitions of acute and chronic UE injury were provided in the survey, participants may have misclassified their injury. While the survey method of data collection is common in racket/paddle sport-related injury studies, no medical provider confirmed the presence or absence of injury. Despite a comparable number of participants in this study with other racket/paddle sport-related injury, the sample size limits generalization.

## 5. Conclusions

Among recreational PB players, the risk of UE injury is higher for those who play more than 2 h a session, play more than three days a week, and play on consecutive days. Additionally acute UE injuries are more likely when using a tight grip for baseline shots while chronic UE injuries are more common in individuals who have been playing for two or more years, play at a higher skill level, and play over six hours per week.

## Figures and Tables

**Table 1:** Frequency data.

Parameter	Category	N	%
Sex	Female Male Prefer not to say	137 116 0	54.2 45.8 0
Age	18–34 35–64 ≥65	57 128 68	22.5 50.6 26.9
Participants with at least one upper extremity injury	No Yes	149 104	58.9 41.1
Participants with at least one upper extremity acute upper extremity injury	No Yes	226 27	89.3 10.7
Participants with at least one chronic upper extremity injury	No Yes	158 95	62.5 37.5
Years of play	<2 ≥2	113 140	44.7 55.3
Skill level	1.0–2.0 2.5–3.5 ≥4 Don’t know	0 186 63 4	0 73 24.9 1.6
Sessions/week	≤3 ≥4	160 93	63.2 36.8
Session length	≤2 h >2 h	166 87	65.6 34.4
Play volume/week	≤6 h ≥7 h	127 126	50.2 49.8
Play on consecutive days	No Yes	97 156	38 61.7
Dink grip	Sufficient Tight	222 31	87.7 12
Volley grip	Sufficient Tight	148 103	58.4 40.7
Baseline grip	Sufficient Tight	134 119	20.9 18.5
Strengthening	No Yes	120 133	47.4 52.6
Stretching	No Yes	159 94	62.8 37.2

**Table 2:** Reported upper extremity injury: between-group differences.

Variable	Category	No Injury	Injury	Statistic
Sex	Female Male	85 64	52 52	Χ^2^(1) = 1.225, *p* = 0.268
Age	18–34 35–64 ≥65	43 60 46	14 68 22	Χ^2^(2) = 16.235, *p* = 0.0003 * Cramer’s V = 0.253 †
Years of play	<2 ≥2	74 75	39 65	Χ^2^(1) = 3.667, *p* = 0.055
Skill level	≤3.5 ≥4	115 31	71 32	Χ^2^(1) = 3091, *p* = 0.079
Session length	≤2 h >2 h	108 41	58 46	Χ^2^(1) = 7.584, *p* = 0.006 *, phi = 0.173
Sessions/week	≥3 ≥4	105 44	55 49	Χ^2^(1) = 8.148, *p* = 0.004 *, phi = 0.179
Play volume/week	≤6 >6	82 67	45 59	Χ^2^(1) = 3.391, *p* = 0.066
Play on consecutive days	No Yes	67 82	30 74	Χ^2^(1) = 6.733, *p* = 0.009 *, phi = 0.163
Dink grip	Mod Tight	124 22	95 8	Χ^2^(1) = 3.038, *p* = 0.081
Volley grip	Mod Tight	91 55	55 48	Χ^2^(1) = 1.986, *p* = 0.159
Baseline grip	Mod Tight	83 63	47 56	Χ^2^(1) = 3.046 *p* = 0.081
Strengthening	No Yes	66 83	54 50	Χ^2^(1) = 1.429, *p* = 0.232
Stretching	No Yes	72 77	48 56	Χ^2^(1) = 0.155, *p* = 0.734

* Significant difference; † Pairwise comparisons of age groups: 18–34 years vs. 35–64 years, [Χ^2^(1) = 13.039, *p* = 0.003, phi = 0.265], 35–64 years vs. ≥65 years [Χ^2^(1) = 7.716, *p* = 0.005, phi = 0.198]; and 18–34 years vs. ≥65 years [Χ^2^(1) = 0.918, *p* = 0.338].

**Table 3:** Reported acute injury: between-group differences.

Parameter	Category	No Injury	Injury	Statistics
Age (in years)	18–34 35–64 ≥65	53 110 63	4 18 5	Χ^2^(1) = 3.128, *p* = 0.209
Sex	Female Male	120 106	17 10	Χ^2^(1) = 0.946, *p* = 0.331
Years of PB play	<2 ≥2	102 124	11 16	Χ^2^(1) = 0.118, *p* = 0.664
Skill level	≤3.5 ≥4	166 56	20 7	Χ^2^(1) = 0.006, *p* = 0.937
Session length	≤2 h >2 h	154 72	12 15	Χ^2^(1) = 6.003, *p* = 0.014, phi = 0.154
Sessions/week	≥3 ≥4	147 79	13 14	Χ^2^(1) = 2.962, *p* = 0.085
Play volume/week	≤6 >6	117 109	10 17	Χ^2^(1) = 2.094, *p* = 0.148
Play on consecutive days	No Yes	94 132	3 24	Χ^2^(1) = 9.479, *p* = 0.002, phi = 0.194
Dink Grip	Sufficient Tight	197 29	25 2	Χ^2^(1) = 0.660, *p* = 0.417
Volley Grip	Sufficient Tight	136 90	17 13	Χ^2^(1) = 0.963, *p* = 0.405
Baseline Grip	Sufficient Tight	126 100	8 19	Χ^2^(1) = 6.607, *p* = 0.010 phi = 0.162
Strengthening	≤2 >2	105 121	15 12	Χ^2^(1) = 0.800, *p* = 0.371
Stretching	≤2 >2	106 120	14 13	Χ^2^(1) = 0.237, *p* = 0.626

**Table 4:** Chronic upper extremity injury.

Parameter	Category	No	Yes	Statistic
Sex	Female Male	92 66	45 50	Χ^2^(1) = 2.818, *p* = 0.093
Age	18–34 35–64 ≥65	45 65 48	12 63 20	Χ^2^(2) = 15.968, *p* = 0.0003 * Cramer’s V = 0.251 †
Years of Play	<2 ≥2	102 79	34 61	Χ^2^(1) = 4.847, *p* = 0.028 *, phi = 0.138
Skill level	≤3.5 ≥4	123 32	63 31	Χ^2^(1) = 4.710, *p* = 0.030 *, phi = 0.138
Session length	≤2 h >2 h	113 45	53 42	Χ^2^(1) = 6.506, *p* = 0.011 *, phi = 0.160
Sessions/week	≥3 ≥4	112 46	48 47	Χ^2^(1) = 10.579, *p* = 0.001 *, phi = 0.204
Play volume	≤6 >6	88 70	39 56	Χ^2^(1) = 5.089, *p* = 0.024 *, phi = 0.142
Play on consecutive days	No Yes	70 88	27 68	Χ^2^(1) = 6.331, *p* = 0.012 *, phi = 0.158
Dink grip	Mod Tight	136 22	86 9	Χ^2^(1) = 1.093, *p* = 0.296
Volley grip	Mod Tight	100 58	50 45	Χ^2^(1) = 2.793, *p* = 0.095
Baseline grip	Mod Tight	88 70	46 49	Χ^2^(1) = 1.260 *p* = 0.262
Strengthening	No Yes	87 78	46 42	Χ^2^(1) = 1.050, *p* = 0.306
Stretching	No Yes	78 80	42 53	Χ^2^(1) = 0.633, *p* = 0.426

* Significant difference, † pairwise comparisons of age groups: 18–34 years vs. 35–64 years, [Χ^2^(1) = 12.979, *p* = 0.0003, phi = 0.265]; 35–64 years vs. ≥65 years [Χ^2^(1) = 7.136., *p* = 0.008, phi = 0.191]; and 18–34 years vs. ≥65 years [Χ^2^(1) = 1.138, *p* = 0.286].

**Table 5:** Relative risk for injury.

	Variable	Relative Risk	Confidence Interval (95%)
Upper extremity injury	Session length (≤2 vs. >2 h)	1.51	1.136–2.017
	Sessions/week (≤3 vs. ≥4)	1.53	1.149–2.044
	Consecutive days	1.53	1.091–2.155
Acute upper extremity injury	Session length (≤2 vs. >2 h)	2.38	1.169–4.868
	Consecutive days	4.97	1.539–16.080
	Baseline grip (Sufficient vs. Tight)	2.67	1.116–5.088
Chronic upper extremity injury	Years of Play (<2 vs. ≥2)	1.45	1.032–2.031
	Skill level (≤3.5 vs. ≥4)	1.45	1.053–2.003
	Session length (≤2 vs. >2 h)	1.55	1.111–2.169
	Sessions/week (≤3 vs. ≥4)	1.68	1.234–2.298
	Play volume (≤ vs. >6 h)	1.45	1.045–2.005
	Consecutive days	1.57	1.085–2.260

## Data Availability

The original contributions presented in this study are included in the article. Further inquiries can be directed to the corresponding author.
